# How does people-centered integrated care in medical alliance in China promote the continuity of healthcare for internal migrants: The moderating role of respect

**DOI:** 10.3389/fpubh.2022.1030323

**Published:** 2023-01-04

**Authors:** Hao Zhang, Yan Wu, Wei Sun, Wuge Li, Xianhong Huang, Tao Sun, Mengjie Wu, Zhen Huang, Shanquan Chen

**Affiliations:** ^1^Department of Health Policy and Management, School of Public Health, Hangzhou Normal University, Hangzhou, China; ^2^Department of Clinical Medicine, School of Clinical Medicine, Hangzhou Medical College, Hangzhou, China; ^3^Faculty of Epidemiology and Population Health, London School of Hygiene and Tropical Medicine, London, United Kingdom

**Keywords:** continuity, people-centered integrated care, medical alliance, China, internal migrant

## Abstract

**Background:**

Continuity is crucial to the health care of the internal migrant population and urgently needs improvements in China. Chinese government is committed to promoting healthcare continuity by improving the people-centered integrated care (PCIC) model in medical alliances. However, little is known about the driving mechanisms for continuity.

**Methods:**

We created the questionnaire for this study by processes of a literature research, telephone interviews, two rounds of Delphi consultation. Based on the combination of quota sampling and judgment sampling, we collected 765 valid questionnaires from developed region and developing region in Zhejiang Province. Structural equation models were used to examined whether the attributes of PCIC (namely coordination, comprehensiveness, and accessibility of health care) associated with continuity, and explored the moderated mediating role of respect.

**Results:**

The result of SEM indicated that coordination had direct effect on continuity, and also had mediating effect on continuity via comprehensiveness and accessibility. The hierarchical linear regression analysis showed that the interactive items of coordination and respect had a positive effect on the comprehensiveness (β = 0.132), indicating that respect has positive moderating effect on the relationship between coordination and comprehensiveness. The simple slope test indicated that in the developed region, coordination had a significant effect on comprehensiveness for both high respect group(β = 0.678) and low respect group (β = 0.508). The moderated mediation index was statistically significant in developed areas(β = 0.091), indicating that respect had moderated mediating effect on the relationship between coordination and continuity via comprehensiveness of healthcare in the developed region; however, the moderated mediation effect was not significant in the developing region.

**Conclusion:**

Such regional differences of the continuity promoting mechanism deserve the attention of policy-makers. Governments and health authorities should encourage continuity of healthcare for migrants through improving the elements of PCIC—coordination, comprehensiveness and accessibility of healthcare, shaping medical professionalism of indiscriminate respect, and empowering migrants to have more autonomy over selection of services and decisions about their health.

## 1. Introduction

Internal migration is defined as human movement within a geopolitical unity. It is a common phenomenon in Europe, the US, and China (1). Evidence shown that internal migration could contribute to poverty reduction, achievements of the Millennium Development Goals (MDGs) and economic growth in developing countries (2). China, the largest developing country, seems to have the most extensive internal migration today (3). As a result of China's reform and opening-up, millions of Chinese farmers have migrated from their home villages and towns to cities that formed one of the largest internal migrations in recent Chinese history (4, 5). According to China's seventh national census, by the end of 2020, internal migrants amounted to 375.82 million, roughly 26.58% of the total population in China, which is considerably larger than most other social groups (6). Internal migration is considered a critical driving force in many sectors including agriculture, manufacturing, construction and services, thus the health of the migrants underpins the productivity of these sectors.

Evidence from internal migration in China revealed that these migrants risk multiple health problem, and continuity of healthcare may provide a crucial solution of the issue. Facing a different set of stressors from non-migrants, such as high-frequency relocation, high risks, low social economic status, and information isolation, the migrants are vulnerable to a wide range of health risks, including communicable and sexually transmitted diseases, occupational injuries and diseases, regeneration health problem and high maternal mortality (7, 8). There is a huge challenge to improve the migrants' health condition, fortunately, previous studies proposed that continuity of healthcare could optimize the health outcomes of migrants. Continuity of healthcare refers to the degree to which a series of discrete healthcare events are experienced as coherent and connected and consistent with the patient's healthcare needs and personal context (9). Continuity may overcome the challenges posed by migration, such as poor communication, information isolation, fragmented treatment and public healthcare (10). There are three aspects of continuity that includes relationship, management, and information. any aspect of continuity, regardless of the situation, can enhance the standard of care (9).

However, the continuity of healthcare for internal migrants calls for significant improvements in China, Firstly, the management continuity of internal migrants, such as the continuous public healthcare and medical service management, is relatively poor. For instance, in the past 20 years, the average full-dose coverage for tuberculosis, diphtheria, pertussis, tetanus, poliomyelitis, measles, and hepatitis B in the immigrant children was estimated to be <60% in the Pearl River Delta region of Guangdong Province, much lower than their non-migrants counterparts (11). Secondly, the relationship continuity with healthcare provider for internal migrants is unsatisfactory. Zeng et al. proposed that migrants considered healthcare professionals from primary care institutions in their hometown to be more knowledgeable about their health situation, which corroborated that the patient-doctor relationship continuity should be improved in migrants' destinations (12). Zheng et al. also suggested that to improve the relationship of family physicians and internal migrants is an tough issue to face (13). Thirdly, the informational continuity of internal migrants is insufficient. Multiple internal migration management departments collect migrants' health information according to their specific management needs, without centralized platform to consolidate the information, resulting in the lack of continuous and consistent information needed in medical services and public healthcare (14).

In order to improve the continuity of healthcare, the Chinese government has been committed to establishing medical alliances for providing people-centered integrated care (PCIC). Evidence shown that the poor healthcare continuity for the migrants, on the one hand, caused by common characteristics of migration such as relocation, isolation and marginalization (15); and on the other hand, the fragmentation of healthcare (16). Deborah Schra (17), Anthony Shih (18) and other scholars all believed that the root cause of the overall poor performance of the healthcare system is fragmentation. To deal with the issue, a PCIC model in medical alliances has been developed in China. PCIC, which was promoted by WHO and jointly advocated by many health systems worldwide, refers to an innovative mode of integrated healthcare around the health needs of population (19). According to the PCIC frame proposed by WHO, the two core approaches of PCIC are the integration and the people-centeredness (20). First of all, an organizational integration of different providers with measures of multidisciplinary coordination, multiple resources accessibility, comprehensive services providing, ect., may provide the bedrock of integrate for PCIC (21). Meanwhile, people-centeredness with a focus on responsiveness, that consciously adopts the individuals' as participants in, and beneficiaries of, trusted health systems, is another key approach of PCIC (20, 22). Respect is regarded as an important dimension of responsiveness which may play a key role for vulnerable groups welfare (23). In China, the medical alliance had been the main platform of PCIC, making effort to improve coordination, comprehensiveness, accessibility and respect, it may have some effect on continuity of healthcare for migrants.

Nowadays, China has taken steps in practicing PCIC in medical alliance. In the years of 2013, the Chinese Health Work Conference formally proposed to explore the establishment of medical alliances, from then on, medical alliances began to blossom across China (23). By 2021, more than 15,000 medical alliances had been established in 205 cities or counties (24). Above of all, these medical alliances were formed by tertiary hospitals, secondary hospitals, and primary institutions, with the horizontal or vertical integration measures of multidisciplinary collaboration, two-way referral, remote medical consultation (25), ect., Luohu, Anhui, Beijing and Zhejiang are typical mature medical alliances (24). Besides, a range of comprehensive services have been provided by multiple providers, with a combination of diagnosis, treatment, nursing, precausion, propaganda, and other services to provide diverse physical, psychological and social support. For instance, the chronic disease joint clinic in medical alliances of Hangzhou City provide a strong example of such integration (26). Meanwhile, individuals have had access to optimized devices, tests and examinations previously scarce at the primary institutions, through inter-institution sharing of resources and information; as well as access to better services by the down-allocation of skilled health personnel from hospitals to primary institutions (27, 28). In addition, respect have also been a part of the PCIC practicing in medical alliances, which establish face-to-face communication channels or online communication platforms to enhance provider-demander interaction and foster shared decision-making (10).

It seems that continuity of healthcare for migrants has been improved with the practice of PCIC in medical alliances, however, the mechanism behind it is unclear. For instance, in 2017, the Ganzhou Fifth People's Hospital Medical Alliance carried out promotion and education on standardized diagnosis and treatment of tuberculosis, resulting in 30% increase in the early detection rate of tuberculosis (29). Similarly, full-dose coverage of vaccination among children of migrant workers increased from 54.28 to 95.21% as a result of health education and follow-up services provided by community healthcare institutions in Beijing (30). The experience of Baoshan Community in Shanghai has proven that the diabetes care home model can effectively enhance health awareness, treatment follow-through and quality of life of diabetic patients among migrants (31). Previous studies proposed that the PCIC may improve continuity of healthcare for migrants (32, 33). We assume that, when accepting healthcare in medical alliances, migrants would have access to consistent medical services, well-connected information, long-term healthcare programs, trusting doctor-patient relationships, however, this assumption requires further verification. Getting insights into the factors contribute to the continuity of healthcare for migrants in the medical alliances context, may ultimately enhance the health status of migrants, and consequently improve social productivity. In this study, we measured the continuity of healthcare among migrants in Zhejiang, China, and analyzed the mechanism driving the continuity of healthcare, by PCIC in medical alliances., Our specific research questions were as follows: (1) What is the influential mechanism of the integration approach–coordination, comprehensiveness and accessibility on continuity? And, (2) what is the influential mechanism of the people-centredness approach–respect on the continuity?

## 2. Theoretical basis

### 2.1. Coordination of healthcare and continuity of healthcare

Coordination of healthcare means a proactive approach to bringing together care professionals and providers, to meet the needs of service users, to ensure that they receive integrated, person-focused care across various settings (10, 34). Recent studies suggest that coordination may influence continuity. For instance, Kuang proposed that coordination ensures that the general practice system to be an open and cooperative system, which establishes organic connection with the secondary and tertiary medical systems and other social service systems, so that patients can smoothly receive continuous diagnosis and treatments provided by different providers to reduce fragmented care and improve continuity (35). Wang proposed that the management of care components in accordance with the patient's demands throughout time and the development of dependable communication channels to support continuity are related to the coordination by the care team (36). Given that numerous empirical research have shown a connection between coordination of healthcare and continuity of healthcare, we propose the following hypothesis.

*Hypothesis 1*. Coordination of healthcare is positively related to continuity of healthcare.

### 2.2. The mediating roles of comprehensiveness of healthcare between coordination and continuity

Comprehensiveness of healthcare is refer to the degree to which the healthcare providers understand and address the vast majority of their physical and common mental healthcare needs, including medical treatment, prevention and health propaganda of acute, chronic, and comorbid conditions (37). Previous studies shown that improved coordination is linked to comprehensiveness, presumably through information sharing, provider communication, and provider cooperation to develop a comprehensive healthcare plan (38). For instance, coordination might be necessary for who have multiple diseases because they usually have complex healthcare needs and require services from different providers (39). Evidence proved that coordination could help patients with multiple chronic diseases in accessing thorough and prompt healthcare services (40). Additionally, collaboration among healthcare professionals that could coordinate the comprehensive healthcare needs of individuals and avoid undertreatment of illnesses while solving competing of different providers (41).

In addition, comprehensiveness is positively related to continuity. Comprehensiveness strives to enhance health outcomes while effectively utilizing healthcare resources by enhancing the quality and continuity of healthcare (42). For instance, a family physician's role as a “gatekeeper” provides an choice for comprehensive care, so that individuals have to visit their doctors several times a year for multiple healthcare needs, which offer opportunities over time for increasing interpersonal continuity through communication (43). Kuang also proposed that general practitioners can implement comprehensiveness of healthcare integrating therapeutic and preventive services so that provide the basis for continuity in health management (44).

Integrating these findings, coordination of healthcare could positively influence continuity of healthcare through comprehensiveness of healthcare. In addition, the following hypotheses are proposed by this study:

*Hypothesis 2*. Coordination of healthcare has positive effects on comprehensiveness of healthcare.

*Hypothesis 3*. Comprehensiveness of healthcare has positive effects on continuity of healthcare.

### 2.3. The mediating roles of accessibility of healthcare between coordination and continuity

Access is the chance to recognize healthcare requirements, seek out healthcare services, locate, receive, or utilize healthcare services, as well as to actually have those needs met (45). Prior research has suggested that coordination of healthcare, which calls for collaboration from management, professionals, and society, can have positive effects on accessibility (46). For instance, Chandni proposed that interprofessional collaboration have a positive impact on individuals' accessibility of healthcare through culturally appropriate information so that led to better understanding and increased utilization of healthcare services (47). Yao et al. found that coordination of healthcare institutions, which encourage hospitals to support primary institutions with healthcare resources and collaborative health program may enable residents to gain high-quality healthcare equipment in grass-root from the higher level hospital and get the consultation of senior specialists (48).

In addition, evidence also proved that accessibility are prerequisite for continuity. Kuang et al. proposed that accessibility guarantees first contact which promote long-term stable doctor-patient relationship and the resulting mutual trust and cooperation (49). Li also proposed that interprofessional collaboration and two-way referral enable patients gain access to the green channel provided by hospital, so that reduce the segments of waiting time, and promote coherent information and health services (50). Gulnaz et al. proposed that poor continuity is usually associated with missing the opportunities for health promotion and disease prevention (51).

Meanwhile, there are also researches proved that accessibility can promote comprehensiveness as well. According to Brennan et al., in the care of heart disease, well-coordinated team may improve patients' access to multidisciplinary specialists, ehabilitation equipment, nutrition guidance courses, doctor-patient interaction platform, which may further promote the comprehensiveness of treatment and health interventions, including diagnosis, treatment, rehabilitation, and prevention (52).

Integrating these findings, coordination may have a positive effect on continuity through accessibility, and such positive effect may also occur through distal mediating effects of accessibility and comprehensiveness. As a result, the following hypotheses are proposed by this study:

*Hypothesis 4*. Coordination of healthcare has positive effects on accessibility of healthcare.

*Hypothesis 5*. Accessibility of healthcare has positive effects on continuity of healthcare.

*Hypothesis 6*. Accessibility of healthcare has positive effects on comprehensiveness of healthcare.

### 2.4. The moderating role of respect

Respect is essential to all human interactions and in the healthcare setting, it permits a certain degree of patient dependency without raising concerns about mistreatment or abuse. Respect for autonomy, as well as for one's own dignity, integrity, and vulnerability, are a few of the characteristics of respect that patients have endorsed (53). Evidence proved that respect can influence whether a patient heeds advice or postpones necessary treatment since it fosters favorable perceptions in the doctor-patient relationship. According to Blanchard et al., individuals who reported being treated disrespectfully were less likely to follow medical advice, undergo a routine physical examination, or receive the proper secondary preventive care for diabetes, heart disease, and hypertension (33). Koskenniemi have found that a strong positive correlation between perceived respect and satisfaction of comprehensiveness of care. According to Tickle et al., continuity—including the number of antenatal visits and post-natal contacts—was directly connected with the level of respect that women reported (54). We suggest that respect can boost the positive relationships between coordination and comprehensiveness, and further enhance the positive relationship between coordination and comprehensiveness.

**Figure 1:**
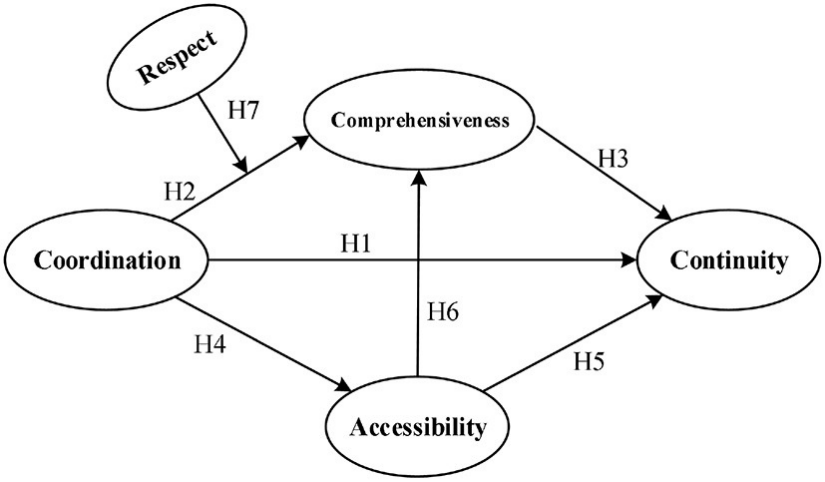
Research model diagram of medical alliances healthcare services quality.

In this research, we first examined the effects of coordination, comprehensiveness, accessibility of healthcare on continuity of healthcare, including direct and indirect effects. Then, we also examined the moderating effects of respect in the links between coordination, comprehensiveness and continuity. The hypothesized model is illustrated in Figure 1.

## 3. Materials and methods

### 3.1. Participants and data collection

The data used in this study were collected from Zhejiang Province, China, for two reasons: first, Zhejiang has a large number of migrants, totaling 25.5 million (55); second, Zhejiang, located in East China, has the largest number of medical alliances, accounting for 40% of the national total, which makes it a suitable location for this study. With a per capita GDP of 113,000 yuan in 2021, Zhejiang Province has an advanced economic status. The government has strived to explore measures to improve the migrants' health and promote health equity by providing adequate rights and health resources to the migrants, including basic medical insurance and work-related insurance, maternal and child healthcare projects, infectious disease precaution services, and health education. Participation in this study was voluntarily and the criteria of including participants in the analysis were as follows: (1) provided informed consent; (2) lived in Zhejiang Province and away from household registered location for more than 6 months; (3) had some experience or understanding of health services provided in the medical alliances; and (4) over 15 years old. The exclusion criteria of participant were as follows: (1) household registered location was within the same municipal jurisdiction as migrant's current home address; (2) separation from household registered location was no more than 6 months; (3) students and temporary residents in hotels, hospitals, airports, and railroad stations.

Sampling areas were chosen using an approach that combines quota sampling with judgment sampling based on level of economic development. Counties in Zhejiang Province were stratified into developed and developing regions through systematic clustering analysis, taking into consideration regional economic indicators (such as gross domestic product, total retail sales of consumer products, etc.), public finance indicators (such as total fiscal revenue, general public budget revenue, etc.) and medical and health services indicators (such as the number of medical institutions, the number of hospital beds, the number of health technicians, etc.), as well as indicators of resident income level that affect the utilization of medical and health services (such as household deposit balance at the end of the year, average disposable income of urban residents, average net income of rural residents, etc.).

Based on the above analysis, the developed region include Hangzhou and Ningbo and the developing region include the remaining counties in Zhejiang Province. We selected the questionnaire respondents using a proportionate quota method based on the proportion of the number of migrants in each region to the total migrants of Zhejiang Province. In this study, Hangzhou was selected as the representative county of the developed region, while Huzhou, Jiaxing and Lishui were selected to represent the developing region. Convenience sampling was then used to select 11 communities in Hangzhou, and 16 communities in the three representative counties in the developing region. Then, 30 representative samples were selected from migrants in each community based on geography, age, gender, and education. Accordingly, 810 questionnaires were distributed to migrants between July 1 and November 30, 2021, of which 765 qualifying questionnaires were retrieved, including 301 from developed areas and 464 from developing areas. Face-to-face interviews were conducted to gather the data after individuals were briefed on the goals of the study, provided their consent and received all relevant explanations. The criteria for excluding questionnaires from the study include: (1) inadequate answers; (2) identical answers to more than half of the questions that were answered; and (3) similar answers to obviously contradictory questions. A questionnaire would be excluded if it met any one of these criteria.

### 3.2. Measurement

The following processes were used to create the questionnaire for this study: (1) through a literature research, items evaluating indications of coordination, accessibility, continuity, comprehensiveness, and respect were selected; (2) telephone interviews were conducted with 107 residents, the interview records were coded and word frequency analysis was performed in NVIVO (version 11) according to the grounded theory; (3) two rounds of Delphi consultation were also conducted with 22 scholars, medical personnel or health administrators to inform the calculation of the average scores of importance and availability of each dimension and the screening and optimization of indicators; (4) A pre-survey was used to further modify and validate the questionnaire. The reliability of the questionnaire was assessed using internal consistency (Cronbach's alpha) and composite reliability (CR). And the measurement's validity was assessed using convergent validity and the heteroplasm-elemental ratio. The questionnaire included the following sections: general information, coordination scale, comprehensiveness scale, accessibility scale, respect scale and continuity scale.

#### 3.2.1. General information

The general information section collected details about respondents' gender, age, marital status, level of education, occupation, income, and place of residence.

#### 3.2.2. Coordination

The coordination scale aimed to evaluate vertical and horizontal collaboration and resource sharing among different healthcare providers. The initial indicators were extracted from literature, including the *Assessment of Interprofessional Team Collaboration Scale* (AITCS) developed by Carole Orchard (56), the findings of Giannoula Tsakitzidis (57) and the *Primary Care Assessment Too*l (PCAT) (58, 59). To optimize the indicators, we also extracted indicators through thematic analysis, with a total word frequency of 322. The indicators were vested through the two rounds of Delphi consultation, with an average score for importance over 4 and an average score for availability above 3.5. The indicators had two components: the vertical coordination and the horizontal coordination (3 items), a seven-point Likert scale was used to measure each item, with higher scores indicating more coordination. The scale ranged from 7 (total agreement) to 1 (total disagreement). The coordination scale's Cronbach's α coefficient was 0.788, its CR value was 0.829, and its AVE value was 0.620. When compared to other scales, the heteroplasm-elemental ratio was <0.90, demonstrating the scale's high reliability and validity (Tables 1, 2).

**Table 1:** Measurement items and results of reliability and validity analysis of the questionnaire (*N* = 765).

**latent variable**	**Measurement items**	**Load^a^**	**Cronbach's α**	**CR**	**AVE**
Coordination	A1 Different medical staff provide coordinate health services for me (the two-way referral, telemedicine, ect.)	0.757	0.788	0.829	0.62
	A2 Hospitals and primary healthcare institutions work together to provide healthcare services.	0.867			
	A3 Medical institutions, public health institutions, volunteer groups and communities work together to provide health services	0.731			
Accessibility	B1 I have access to the healthcare devices and medical equipment I need.	0.69	0.783	0.789	0.485
	B2 I can get the medicine I need	0.709			
	B3 I have access to the services that are friendly to my culture and values	0.622			
	B4 I have access to the health services I need.	0.752			
Comprehensiveness	C1 My mental health and emotional feelings are concerned by healthcare. Medical staff monitor your mental state when providing you with medical and health services.	0.907	0.843	0.856	0.673
	C2 My life needs and social function are concerned by healthcare. Medical staff pay attention to your life confusion when providing you with medical and health services.	0.921			
	C3 My diverse physiological needs are concerned by healthcare.	0.589			
Continuity	D1 Medical staff have a comprehensive understanding of me (health status history, life history, etc.).	0.747	0.719	0.735	0.481
	D2 Different providers provided consistent care at different stages (follow-up, long-term treatment, etc.)	0.697			
	D3 My health information is coherent and can be transmitted and shared among different institutions, departments, and medical staff.	0.632			
Respect	E1 My life and personal dignity is respected.	0.769	0.83	0.834	0.503
	E2 My rights to choose medical institutions, medical staff and treatment options is respected.	0.756			
	E3 My rights of health privacy is respected and protected.	0.716			
	E4 The medical staff listened patiently to my medical and health service needs and feelings.	0.608			
	E5 The medical staff explained to me clearly.	0.686			

a All load values are significant at the 0.001 level; CR, composite reliability; AVE, average variance extraction.

**Table 2:** Heterotrait—Monotrait (HTMT) of the questionnaire (*N* = 765).

**Latent variable**	**Coordination**	**Accessibility**	**Comprehensiveness**	**Continuity**	**Respect**
Coordination	–				
Accessibility	0.624	–			
Comprehensiveness	0.699	0.619	–		
Continuity	0.769	0.676	0.701	–	
Respect	0.368	0.610	0.437	0.482	–

#### 3.2.3. Comprehensiveness

The comprehensiveness scale aimed to evaluate whether migrants think that healthcare professionals can provide them with integrated services, mainly including physiological, psychological and social aspects. The initial indicators were extracted from literature, including the *Chiba Inter-Professional Cooperation Competency Scale* (CICS29) (60), *Inter-Professional Educational Collaboration Framework* (IPEC) (61), the *Assessment of Interprofessional Team Collaboration Scale* (AITCS) (56) and the *Primary Care Assessment Tool* (PCAT) (58, 59). To optimize the indicators, we also extracted indicators through thematic analysis, with a total word frequency of 222. The indicators were vested through the two rounds of Delphi consultation, with an average score for importance over 4 and an average score for availability above 3.5. The indicators had three components: physiological, psychological and social perspectives (3 items), a seven-point Likert scale was used to measure each item, with higher scores indicating more comprehensiveness. The scale ranged from 7 (total agreement) to 1 (total disagreement). The comprehensiveness scale's Cronbach's α coefficient was 0.843, its CR value was 0.856, and its AVE value was 0.673. When compared to other scales, the heteroplasm-elemental ratio was <0.90, demonstrating the scale's high reliability and validity (Tables 1, 2).

#### 3.2.4. Accessibility

The accessibility scale aimed to evaluate migrants' access to medical and health services, including the accessibility of services and resources and culture acceptance. The initial indicators were extracted from literature, including the *Chiba Inter-Professional Cooperation Competency Scale* (CICS29) (60), *Inter-Professional Educational Collaboration Framework* (IPEC) (61), the *Assessment of Interprofessional Team Collaboration Scale* (AITCS)(56) developed by scholars from Japan, Canada and the United States and the *Primary Care Assessment Tool* (PCAT) (58, 62). To optimize the indicators, we also extracted indicators through thematic analysis, with a total word frequency of 261. The indicators were vested through the two rounds of Delphi consultation, with an average score for importance over 4 and an average score for availability above 3.5. The indicators had three components: service accessibility, resource accessibility and cultural acceptance (4 items), a seven-point Likert scale was used to measure each item, with higher scores indicating higher accessibility. The scale ranged from 7 (total agreement) to 1 (total disagreement). The accessibility scale's Cronbach's α coefficient was 0.783, its CR value was 0.789, and its AVE value was 0.485. When compared to other scales, the heteroplasm-elemental ratio was <0.90, demonstrating the scale's high reliability and validity (Tables 1, 2).

#### 3.2.5. Continuity

The continuity scale aimed to evaluate the degree of service coherence received and experienced by migrants, including the continuity of interpersonal interaction, services and resources. The initial indicators were extracted from literature, including the findings of Reeves S (63), and the importance of “building a unified information platform” mentioned by several documents of the National Health Commission of China (64, 65). To optimize the indicators, we also extracted indicators through thematic analysis, with a total word frequency of 129. The indicators were vested through the two rounds of Delphi consultation, with an average score for importance over 4 and an average score for availability above 3.5. The indicators had three components: interpersonal continuity, service continuity and resource continuity (3 items), a seven-point Likert scale was used to measure each item, with higher scores indicating better continuity. The scale ranged from 7 (total agreement) to 1 (total disagreement). The continuity scale's Cronbach's α coefficient was 0.719, its CR value was 0.735, and its AVE value was 0.481. When compared to other scales, the heteroplasm-elemental ratio was <0.90, demonstrating the scale's high reliability and validity (Tables 1, 2).

#### 3.2.6. Respect

The respect scale aimed to evaluate migrants' perception of respect and privacy protection, etc. in the process of the treatment. The initial indicators were extracted from literature, including three components proposed for measuring human respect in the reactivity scale: autonomy, dignity and confidentiality in *The World Health Report, 2000* (66, 67) and communication as an additional aspect of respect from: *Multi-country Survey Study on Health and Health System's Responsiveness in 60 different countries in 2000-2001* (MCSS) (68, 69) developed by WHO. To optimize the indicators, we also extracted indicators through thematic analysis, with a total word frequency of 82. The indicators were vested through the two rounds of Delphi consultation, with an average score for importance over 4 and an average score for availability above 3.5. The indicators had four components: autonomy, dignity, confidentiality and communication (5 items), a seven-point Likert scale was used to measure each item, with higher scores indicating more respect. The scale ranged from 7 (total agreement) to 1 (total disagreement). The respect scale's Cronbach's α coefficient was 0.830, its CR value was 0.834, and its AVE value was 0.503. When compared to other scales, the heteroplasm-elemental ratio was <0.90, demonstrating the scale's high reliability and validity (Tables 1, 2).

### 3.3. Quality control

We conducted a pilot study before collecting the data used in the final analysis. After gathering and analyzing the issues found in the pilot study, we made revisions to the questionnaire and defined a clear study strategy. Investigators were selected from post-graduates with appropriate personal survey experience. Additionally, investigator training was provided before the survey was conducted to make sure that the investigators understood the project, the questionnaire, and the important details of the inquiry as well as adhered to consistent norms and practices, such as: (1) the principle of consistency required that the consistency of investigators' survey techniques should reach more than 95%; (2) the principle of conformity required that no <5% of the respondents be randomly selected for repeated investigation, and the coincidence rate of repeated investigation should reach 97%; (3) after the survey, the accuracy of the data was checked in time, and the incorrect and missing items in the questionnaire should be supplemented by a telephone return visit.

### 3.4. Ethical considerations

Before data collection, participants were informed of the study's objectives and its methods. Participants were made aware that taking part was completely voluntary and that their information would be coded to protect their privacy and only be utilized for research purposes. The study was approved by the Ethics Committee of Hangzhou Normal University (approval number: 20190022). The study was carried out in conformity with the ethical guidelines outlined in the 1964 Declaration of Helsinki and its later amendments, and each participant gave their informed consent.

### 3.5. Statistical analysis

#### 3.5.1. Preliminary analyses

Data in this study was analyzed using Amos version 20.0, SPSS version 23.0 and the macro PROCESS procedure for SPSS version 4.1. We used the following criteria to assess normalcy, outliers, and multicollinearity: Kurtosis (ku) and coefficients of skewness (sk) were used to measure normalcy; the existence of outliers was identified by Mahalanobis Distance; the variance inflation factor (VIF) and tolerance rate were used to test for multicollinearity. We also used Spearman's correlation for bivariate associations (reported as values of r) and descriptive statistics for the primary study variables (given as means standard deviations).

#### 3.5.2. Mediation and moderation analyses

The mediating impact between the perceived coordination and perceived continuity of healthcare services was examined using structural equation modeling (SEM) through accessibility or comprehensiveness. The reliability of the questionnaire was assessed using composite reliability (CR) and internal consistency (Cronbach's coefficient). Convergent validity (Average Variance Extracted, AVE) and discriminant validity (Heterotrait—Monotrait, HTMT) were used to assess the measurement's validity. To estimate and verify the acting path from coordination, accessibility, comprehensiveness, to continuity, we conducted the maximum-likelihood method in the SEM. To test the mediating effect, we used 5,000 repeats of the percentile bootstrap approach at a 95% confidence level (CI). The difference in effect was regarded as statistically significant if it did not include 0. To test the moderating effect of respect on the relationship between coordination and comprehensiveness, we used the hierarchical linear regression analysis with SPSS. Finally, in order to further investigate the influence respect has on the link between coordination and continuity through comprehensiveness, a straightforward slope test was conducted. The conditional indirect influence of coordination on continuity through comprehensive was examined when the respect score was at the sample mean, plus 1 SD and minus 1 SD.

## 4. Results

### 4.1. Demographic characteristics

The descriptive analysis is shown in Table 3. Of the 765 samples, 338 (44.2%) were male and 427 (55.8%) were female. The participants were mainly in the age group of <45 years old (76.7%), followed by those in the 45–60 age group (15.8%), and those above 60 years old (7.5%). In terms of marital status, 424 participants (55.4%) were married. As far as education is concerned, 231 (30.2%) had a university degree, i.e., a bachelor's degree or above, and from income perspective, 316 (41.3%) were low-income. In terms of occupation, 133 participants (17.4%) were professionals, 113 participants (14.8%) were business service staff, and 396 (51.8%) were others. Lastly, 301 (39.3%) lived in the developed region while 464 (60.7%) lived in the developing region.

**Table 3:** Comparison of migrants' mean scores on the continuity questionnaire based on different demographic variables (*N* = 765).

**Characteristic**	**Categorization**	**Total score**
Gender	Male (338)	5.41 ± 1.04
	Female (427)	5.14 ± 1.11
	*t* (*p*)	3.38 (<0.01)
Age (years)	<45 (587)	5.23 ± 1.12
	45–60 (121)	5.32 ± 0.95
	≥60 (57)	5.48 ± 1.04
	*F* (*p*)	1.53 (0.21)
Marital status	Unmarried (329)	5.28 ± 1.14
	Married (424)	5.24 ± 1.04
	Divorced (8)	5.88 ± 0.95
	Other (4)	5.26 ± 1.49
	*F* (*p*)	0.93 (0.42)
Educational background	Uneducated (21)	5.51 ± 0.93
	Primary school (53)	5.29 ± 1.05
	Middle school (123)	5.30 ± 1.05
	High school (172)	5.30 ± 1.09
	Junior college (165)	5.32 ± 1.10
	College and above (231)	5.15 ± 1.12
	*F* (*p*)	0.94 (0.46)
Occupation	Leader (79)	5.07 ± 0.98
	Professionals (133)	5.45 ± 1.01
	Office clerk (34)	5.34 ± 0.95
	Business Service staff (113)	5.03 ± 1.06
	Agriculture, forestry, animal husbandry, and fishery personnel (10)	4.94 ± 1.00
	Others (396)	5.31 ± 1.14
	*F* (*p*)	2.76 (0.02)
Income	Low (316)	5.38 ± 1.12
	Middle (97)	5.28 ± 1.13
	High (352)	5.16 ± 1.04
	*F* (*p*)	3.38 (0.04)
Region	Developed area (301)	5.14 ± 1.13
	Developing (464)	5.35 ± 1.05
	*t* (*p*)	−2.61 (<0.01)

Migrants' perceptions of continuity of healthcare were assessed by scale scores. Participants' continuity scores varied significantly across gender (*t* = 3.38, *p* < 0.01), occupation (*F* = 2.76, *p* = 0.02), income (*F* = 3.38, *p* = 0.04) and region (*t* = −2.61, *p* < 0.01). Male migrants (M = 5.41, SD = 1.04) scored higher than female migrants (M = 5.14, SD = 1.11). Low-income migrants (M = 5.38, SD = 1.12) scored higher than high-income migrants (M = 5.16, SD = 1.04). The professional personnel in the migrants had the highest score (M = 5.45, SD = 1.01), and the agricultural, forestry, animal husbandry, and fishery workers had the lowest score (M = 4.94, SD = 1.00). Migrants in less developed regions (M = 5.35, SD = 1.05) scored higher than those in developed regions (M = 5.14, SD = 1.13). Health service continuity scores based on participant demographics is presented in Table 3.

Table 4 shows the average and standard deviation of coordination, accessibility, comprehensiveness, continuity, and respect, and the Pearson correlation coefficients between variables. All the correlations between variables were significant, among which there were significant positive correlations among coordination, accessibility, comprehensiveness, continuity, and respect (*P* < 0.01).

**Table 4:** Mean, standard deviation, and correlation coefficient of each variable (*N* = 765).

**Variable**	**1**	**2**	**3**	**4**	**5**
1. Coordination					
2. Accessibility	0.503^**^				
3. Comprehensiveness	0.577^**^	0.497^**^			
4. Continuity	0.599^**^	0.512^**^	0.547^**^		
5. Respect	0.311^**^	0.498^**^	0.377^**^	0.380^**^	
Mean value	5.278	5.554	4.983	5.265	6.084
Standard deviation	1.0710	0.9025	1.2587	1.0882	0.7945

** p < 0.01, two-tailed test.

### 4.2. Construction and fit of the SEM model

**Figure 2:**
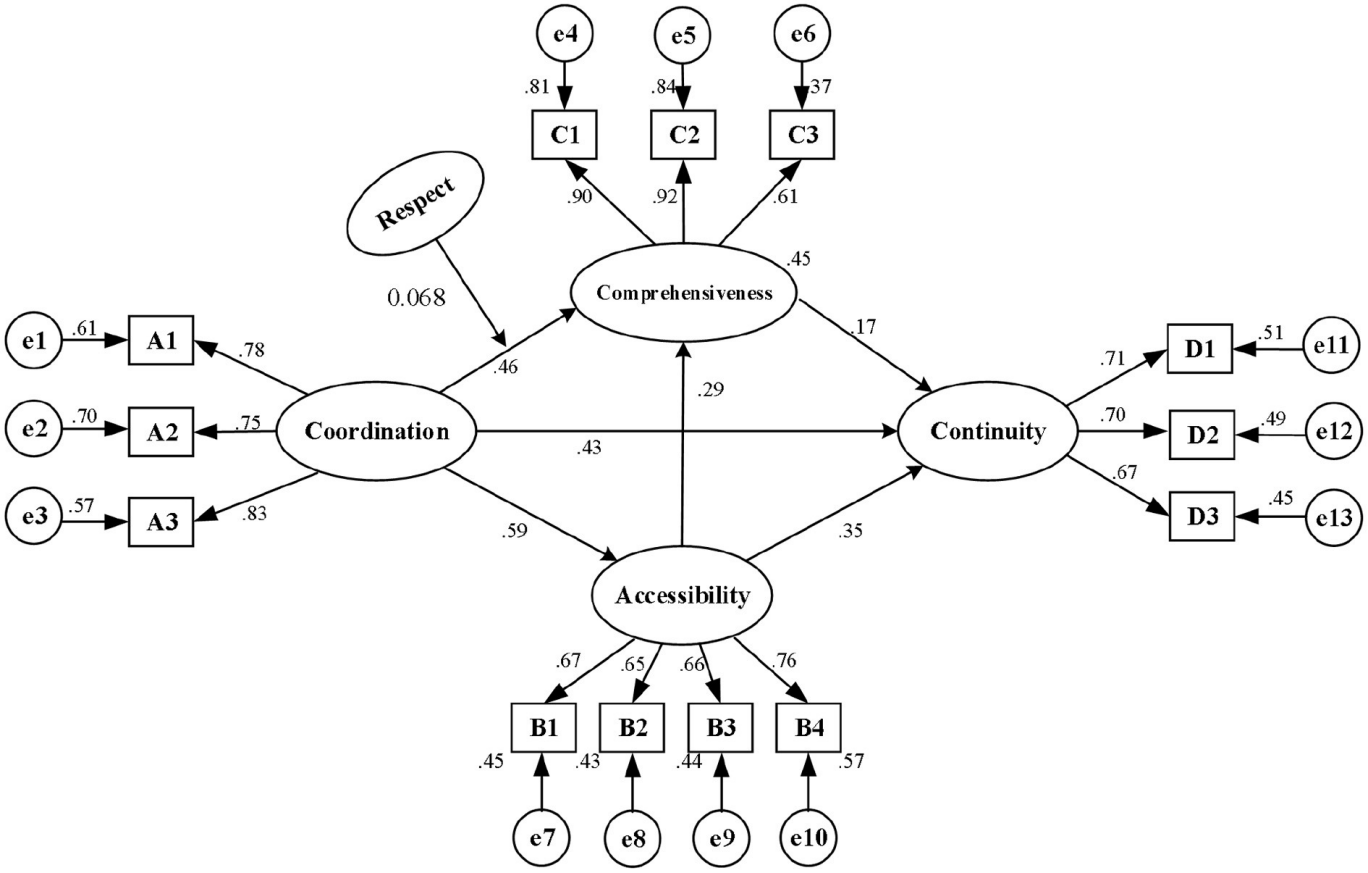
Model diagram of driving mechanism of the medical alliances promoting the continuity of health service for migrants.

In this study, coordination, comprehensiveness and accessibility were taken as independent variables, and continuity was the dependent variable used to build an SEM. The maximum-likelihood method was used to estimate the initial model. The C.R. value in essence represents the normalized estimation of multivariate kurtosis (70). Values >5.00, according to Bentler (71), are typically a sign that the data are non-normally distributed, in practice. The sample in this application had a z-statistic of 61.097, which was suggestive of non-normality. Therefore, in order to accommodate the lack of multivariate normality, the model and parameters were adjusted using the Bollen-Stine bootstrap approach (72). The model was accepted as the results of fitting parameters showed that the *p*-value of each path was <0.05, then the model was modified with correction index. Figure 2 is the exhibition of the ultimate model, with each fitting index showing a good fit as presented in Table 5. Figure 2 presents the standardized path coefficients for the complete causal model. Six of the seven causal relationships hypothesized in the model were found to be statistically significant to different degrees with positive path coefficients: (i) coordination and comprehensiveness; (ii) comprehensiveness and continuity; (iii) coordination and continuity; (iv) coordination and accessibility; (v) accessibility and continuity; (vi) accessibility and comprehensiveness.

**Table 5:** Fitting results of the structural equation model.

**Fitting index**	**Fitting standard**	**Model**	

		**Initial model**	**Modified model**
Chi-square freedom ratio χ^2^/*df*	1 <χ^2^/*df* <3 Good	4.063	1.464
Root mean square error of approximation RMSEA (90%CI)	<0.05 Good	0.063	0.025
Goodness of fit index, GFI	>0.90 Good	0.954	0.981
Adjusted goodness of fit index, AGFI	>0.90 Good	0.930	0.961
Normed fit index, NFI	>0.90 Good	0.947	0.981
Confirmatory fit index, CFI	>0.90 Good	0.960	0.994
Bentler and Bonett's non-normed fit index, TLI	>0.90 Good	0.948	0.992

By standardizing the effects, we found that coordination, comprehensiveness and accessibility had positive effects on continuity, with standardized path coefficients being 0.43, 0.17, and 0.35 (*p* < 0.001), respectively, supporting the hypotheses H1, H3, and H5. Coordination and accessibility contributed positive effects on comprehensiveness, with the standardized path coefficients being 0.46 and 0.29 (*p* < 0.001), respectively, supporting the hypotheses H2 and H6. Additionally, coordination had positive effects on accessibility, with the coefficient being 0.59 (*p* < 0.001), supporting hypothesis H4.

### 4.3. Analysis of simple mediating effect

The simple mediating effect between coordination and continuity was tested by bootstrap, as shown in Table 6. Above all, the indirect effects of coordination on continuity *via* comprehensiveness were significant (the 95% CI was 0.033–0.131), and the direct effects of coordination on continuity was also significant (the 95% CI was 0.283–0.544). Since the 95% CI in the percentile columns did not include 0, it indicated that comprehensiveness partially mediated the effect of coordination on continuity. Besides, both the indirect effect (the 95% CI was 0.065–0.216) and direct effects (the 95% CI was 0.283–0.544) of coordination on continuity *via* accessibility were significant, reflecting that access also partially mediated the effect of coordination on continuity. Ultimately, the distal indirect effects of coordination on continuity *via* accessibility and comprehensiveness were significant (the 95% CI was 0.011–0.051), and the direct effects of this path were also prominent (the 95% CI was 0.283–0.544), validating that accessibility and comprehensiveness incompletely mediates the effect of coordination on continuity.

**Table 6:** Bootstrap confidence interval estimation results of simple mediating effect.

**Variable relationship**	**Effect type**	**Effect value**	**LLCI**	**ULCI**	**Supported hypothesis**
Coordination → comprehensiveness → continuity	Overall effect	0.491	0.366	0.627	H_2_, H_3_
	Direct effect	0.412	0.283	0.544	
	Indirect effect	0.079	0.033	0.131	
Coordination → accessibility → continuity	Overall effect	0.522	0.443	0.661	H_4_, H_5_
	Direct effect	0.412	0.283	0.544	
	Indirect effect	0.140	0.065	0.216	
Coordination → accessibility → comprehensiveness → continuity	Overall effect	0.441	0.314	0.573	H_4_, H_6_, H_3_
	Direct effect	0.412	0.283	0.544	
	Indirect effect	0.029	0.011	0.051	

LLCI represents Lower limit 95% confidence intervals; ULCI represents upper limit: 95% confidence intervals.

### 4.4. Analysis of moderating effect

The hierarchical linear regression analysis was conducted to examine the moderating effect of respect between coordination and comprehensiveness. Model 1 added statistically significant control variables (Table 7) to the univariate analysis. Model 2 added independent variables (coordination) and moderating variables (respect) on the basis of Model 1, and Model 3 added the interactive items of independent variables and moderating variables (coordination^*^respect) on the basis of model 1 and model 2. To avoid multicollinearity between the variables, the independent variable (coordination), the dependent variable (comprehensiveness), and the moderating variable (respect) were centered before testing. The maximum variance expansion factor of the three models is 1.739, which is significantly lower than 10, indicating that no issue of multicollinearity was detected, and the results are reliable.

**Table 7:** Moderating effect of respect on the relationship between coordination and comprehensiveness.

**Variable**	**Model 1**	**Model 2**	**Model 3**
**Gender**			
Male (reference group)			
Female	−0.082^*^	−0.034	−0.03
**A**ge (years)			
Youth (<45) (reference group)			
Middle-aged (<60)	−0.007	−0.014	−0.009
Elderly (≥60)	0.064	0.068	0.078
**M**arital status			
Unmarried (reference group)			
Married	−0.141^**^	−0.082^*^	−0.088^*^
Divorced	0.02	−0.007	−0.006
Others	0.007	0.007	0.006
**E**ducational background			
Uneducated (reference group)			
Primary school	−0.006	0.01	0.008
Middle school	−0.013	0.025	0.029
High school	−0.023	−0.005	−0.007
Junior college	−0.059	−0.012	−0.012
College and above			
**O**ccupation			
Leader (reference group)			
Professionals	0.084^*^	0.078^*^	0.071^*^
Office clerk	0.044	−0.001	−0.009
Business service staff	0.027	0.009	0.008
Agriculture, forestry, animal husbandry and fishery personnel	0.027	0.033	0.029
Others	0.141^***^	0.084^**^	0.081^**^
**I**ncome			
Low (reference group)			
Middle	0.003	0.038	0.041
High	−0.03	0.008	0.014
**R**egion			
Developed area (reference group)			
Developing	0.132^***^	0.052	0.055
Respect		0.473^***^	0.474^***^
Coordination		0.217^***^	0.233^***^
Respect × Coordination			0.068^***^
*R* ^2^	0.091	0.409	0.414
Adjusted *R*^2^	0.069	0.393	0.397
Δ*R*^2^	0.091	0.318	0.005
Variance ratio	4.145	25.719	25.001
Δ*F*	4.145	199.983	6.693
VIF_max_	1.719	1.732	1.739

^*^p < 0.05, ^**^p < 0.01, ^***^p < 0.001; VIF_*max*_, maximum variance expansion factor.

The results of Model 1 showed the influence of the control variable on the dependent variable. Gender, marital status, occupation and region had significant effects on the perceived comprehensiveness of healthcare. Female demonstrated a lower comprehensiveness score than their male counterparts (β = −0.082, *P* = 0.024). Married people perceived a significantly lower comprehensiveness score than unmarried people (β = −0.082, *p* = 0.02). In terms of occupation, technical professionals (β = 0.084, *P* = 0.03) and other personnel (β = 0.141, *P* < 0.001) had better perceived comprehensiveness of healthcare than organizational leaders. In addition, the group in the developing region perceived a higher comprehensiveness score than those in the developed region (β = 0.132, *p* < 0.001). The results of model 2 showed that the main effects of independent variable coordination (β = 0.473, *p* < 0.001) and moderating variable respect (β = 0.217, *p* < 0.001) on comprehensiveness were positive. Model 3 showed that the interactive items of coordination and respect had a positive effect on comprehensiveness (β = 0.132, *p* = 0.01), indicating respect have a positive moderating effect on the relationship between coordination and comprehensiveness.

**Figure 3:**
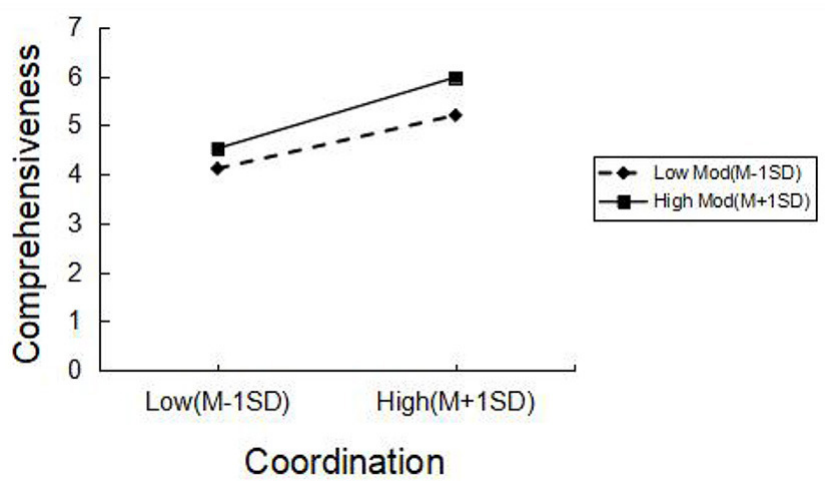
Moderating effect diagram of respect.

The moderating role of respect between coordination and comprehensiveness was further explored by a simple slope test. The results demonstrated that respect had a considerable impact on the correlation between these two variables. The interaction diagram (Figure 3) visually reflects the moderating effect of respect on the relationship between coordination and comprehensiveness. It illustrated the interaction at high (plus 1 SD) and low (minus 1 SD) levels of coordination and respect, and the slope represents the influence of coordination on comprehensiveness. The results suggested that, for migrants who perceived higher level of respect in healthcare, there was a more powerful and positive link between coordination and comprehensiveness compared with those who perceived lower level of respect. Specifically, coordination had a significant effect on comprehensiveness for both high-respect perception group (β =0.678, *t* = 14.675, *p* < 0.001) and low-respect perception group (β = 0.508, *t* =10.499, *p* < 0.001).

### 4.5. Analysis of moderated mediation effects

The indirectly conditional effect of coordination on continuity through comprehensiveness at various values of respect was analyzed when the respect score was the sample mean and plus or minus 1 SD. The results showed that the indirectly conditional effects were significant in the mean, high level (+1 SD) or low level (−1 SD) of the migrants in both developed and developing regions, as supported by the bootstrap 95% CI. The moderated mediation index was statistically significant in the developed region (β = 0.091), while it was not significant in the developing region, since the bootstrap 95% CI included 0 (see Tables 8, 9), indicating that respect had a moderated mediating effect on the relationship between coordination and continuity *via* the comprehensiveness in the developed region.

**Table 8:** Bootstrap confidence interval estimation results of moderated mediating effect: specific conditional values of respect.

	**Developed area**				**Developing area**			
	β	**SE**	**LLCI**	**ULCI**	β	**SE**	**LLCI**	**ULCI**
−1 SD	0.144	0.043	0.067	0.233	0.189	0.04	0.109	0.264
Mean	0.218	0.046	0.133	0.313	0.179	0.03	0.121	0.24
+1 SD	0.292	0.06	0.176	0.413	0.169	0.035	0.107	0.246

LLCI represents Lower limit 95% confidence intervals; ULCI represents upper limit: 95% confidence intervals.

**Table 9:** Index of moderated mediation.

	**Developed area**				**Developing area**			
	**Index**	**SE**	**LLCI**	**ULCI**	**Index**	**SE**	**LLCI**	**ULCI**
Respect	0.091	0.031	0.037	0.157	−0.11	0.026	−0.055	0.048

LLCI represents Lower limit 95% confidence intervals; ULCI represents upper limit: 95% confidence intervals.

## 5. Discussion

### 5.1. Practice implications and future prospects

This study proves the mechanism of the important dimensions of PCIC in medical alliances–continuity, accessibility and comprehensiveness, in promoting the continuity for migrants, and also explored the moderated mediating role of respect. Data collected from 765 migrants in representative counties from Zhejiang Province was analyzed using SPSS, SEM, and PROCESS procedure for SPSS. The result of SEM indicated that coordination had direct effect on continuity, and also had mediating effect on continuity *via* comprehensiveness and accessibility. The hierarchical linear regression analysis indicated that respect have positive moderating effect on the relationship between coordination and comprehensiveness. The simple slope test indicated that in the developed region, respect had moderated mediating effect on the relationship between coordination and continuity *via* comprehensiveness of healthcare.

When analyzing migrants' perceived continuity, we found that the group living in the developing region of Zhejiang Province experienced better service continuity when seeking healthcare. This difference may be a result of primary healthcare institutions in developing areas being more closely connected to migrants. This finding is consistent with previous studies showing that small primary care institutions in rural areas can improve the continuity of provider-patient relationships by increasing healthcare providers' understanding of migrants' needs (12, 73). This research also showed that female migrants had a perception of poorer continuity as compared to their male counterparts. This may be a result that women have higher needs and requirements for service continuity, such as long-term reproductive health guidance, thus are more sensitive to the continuity gaps (74, 75). In addition, participants who were professionals expressed better experience of continuity, the reason may be that they are generally more capable of communicating their needs and understanding care providers' advices, thus received more respect from the care providers. It is consistent with some previous studies that positive communication with providers can lead to better continuity of healthcare (76, 77). The findings may provide an alternative way to improve continuity through PCIC when resource is limited. Among the medical alliances, although those in the developing region may have more limited healthcare resources and less advanced information technology, continuity in this region is still higher than those in the developed region. It demonstrated that, to some extent, soft power such as doctor-patient relationship, attention to patient demand and respect are equally important to the resource investments into facilities and equipment. Gender and occupational differences in perceived continuity further underscore the importance of providing people-centered services to vulnerable groups. Although the socioeconomic status of women has improved significantly worldwide, women remain a vulnerable group. Due to women's educational and economic disadvantages, they need more respect, understanding and support in healthcare services, and more targeted healthcare programs are needed to prioritize long-term maternal and child care. Moreover, as migrants often engage in low SES occupations other than professionals, it requires healthcare providers to take into consideration their less advanced communication skills and special healthcare needs, to design more appropriate services, improve communication skills, and give necessary respect. Nowadays, it seems that there is still a huge gap to bridge.

Coordination, accessibility, comprehensiveness as attributes of healthcare, are widely used to measure the extent of PCIC practice. However, previous studies have not addressed the interrelatedness and the relative importance of these PCIC attribute (78). This study provided some evidence of the mechanisms for the impact of these attributes on continuity. The results of the SEM showed that coordination, comprehensiveness, and accessibility can predict the perceived continuity by migrants, to some extent, and the migrants may experience better care continuity by seeking healthcare with PCIC. The possible mechanisms may be that: firstly, the continuity of service management was improved by comprehensive healthcare services designed to meet diverse needs by inter-professional and across-organizations coordination and coherent service procedures such as two-way referral; secondly, the continuity of information was improved by sharing examination, imaging and other equipment and resources in the medical alliances, providing migrants access to homogeneous health resources and consistent information across suppliers, and; thirdly, the continuity of interpersonal relationships was also improved, as migrants have access to providers' information and doctor-patient contact through e-health platforms to get more familiar with providers and establish long-term relationship with medical staff. It is evident that accessibility and comprehensiveness are important factors mediating the coordination-continuity relationship, which is consistent with existing research on continuity of healthcare. For instance, Yao demonstrated that information sharing and inter-professional collaboration can encourage the flow of medical staff to the grass-roots, further provide comprehensive and coherent service for residents (43, 48), Ke and Wang argued that, the integration of healthcare enables people to gain access to comprehensive service and obtain timely and long-term health (44, 49). The results shown the importance of healthcare integration on continuity, as the attributes of PCIC do not function individually but are linked in a mutually supportive manner. The findings may provide a perspective for the governments, health authorities, providers to further deepen PCIC for higher continuity. Collaboration among providers may provide a cornerstone of PCIC healthcare, thus, further effective measures of creating internal drivers for long-term, broad collaboration should be taken, such as deepen interests and responsibility sharing among different stakeholders. Since accessibility and comprehensiveness are important mediating factors for coordination, in medical alliances, a series of mutually supportive measures should be adopted, such as optimizating collaborative service procedure for convenience of access, designing more people-centered healthcare program around the migrants needs, optimizing resource allocation and effective utilization for comprehensive services, ect., so as to improve the health outcome brought by continuity through PCIC.

Furthermore, the mediation models provided a more detailed explanation of the mechanisms of coordination and continuity. Respect as an important dimension of PCIC was demonstrated to be a factor in the moderating mechanisms on the relationship between coordination and continuity. Interestingly, we found that comprehensiveness has a significant positive mediation effect between coordination and continuity which is moderated by respect in the developed region, while, the result is insignificant in the developing region. The insignificant result in the developing region, was probably because that migrants there were less sensitive to respect, and additionally, respect for individual is relatively undifferentiated in this region. In other words, in the developed region, respect in healthcare is discriminatory to some extent. Respect is a crucial factor that deserves attention from policymakers and healthcare providers. Thus, it is necessary to boost indiscriminate respect for migrants by developing medical ethics and professionalism among medical staff, establishing people-centered cultural environment in medical alliances, promoting clinical practice and typical case discussions to understand patients' right of privacy, informed consensus and autonomy. Moreover, freedom of choice is also an important aspect of respect, and it is necessary for migrants to be educated and empowered to have the right and ability to choose the appropriate services or providers, and make decisions together (79, 80).

Our results also highlighted that we may pay attention to the influence of socio-economic and cultural context of healthcare. Under the traditional healthcare mode in China, a friendly and mutually-respectful doctor-patient relationship was promoted, and the interaction between grass-root doctors and patients formed in the early days of China's liberation is a typical example. However, with modernization, urbanization and marketization, such a close-knit sociocultural is eroding, which may explain why respect for migrants may be worse in the developed region. To some extent, this is a warning that social cultural environment is a key aspect in improving health welfare of migrants. However, it is gratifying that the development of PCIC may shape a new cultural of respect, which empoweres individual value expression and freedom of choice, thus providing the basis for migrants to express free will and satisfy their diverse health needs. In medical alliance, individuals have a growing selection of health services and providers, which may empower them to integrate more self-values into health practice, thus promoting the continuity of relationships and the long-term care utilization, especially in developed region. This finding may have reference for the developing countries with insufficient health resources and unsatisfied health equity. In many region like Asia, although internal migrants have contributed significantly to economic growth and gained from higher wages in higher productivity areas, they remain socially and economically excluded from the wider benefits of economic growth such as continuity of healthcare (81). Although this study had been carried out in China, the integration of healthcare and the respectful environments brought about by PCIC can provide reference by other countries facing similar challenges of internal migrants under similar environment.

### 5.2. Strengths and limitations

Firstly, the comprehensive search of multiple databases ensured that the scales published in academic journals in line with the research content are widely included. And the scale was designed locally through informed interviews and Delphi consultation to ensure that the scale could accurately measure the continuous medical and healthcare services perceived by migrants in China under the background of medical alliances. Secondly, we adopted the method of combining quota sampling with judgment sampling, and selected sample sites based on the level of social and economic development, which enabled us to conduct a comprehensive, detailed and representative analysis of the role of medical alliances in promoting the continuity of services for migrants in Zhejiang Province. Thirdly, through moderated mediation analysis, the mediating effect of coordination and comprehensiveness is proved, and the moderating effect of respect is verified. This study unveiled the impact paths and interaction mechanisms of variables including coordination, accessibility, comprehensiveness and continuity, and the impact mechanism of continuity, as well as the unique role of respect in it, which enriches the existing research on healthcare service continuity of migrants.

The following are possible defects in this study. Firstly, a bias in the selection of research object may exist as participants may have chosen to participate in this study because of their attention to healthcare through medical alliances. Secondly, since data collection used self-report measures, the response bias cannot be ruled out, and the measures were based on individual experience rather than objective data, although we set control variables to eliminate individual interference. Thirdly, since the questionnaire adopted in this research was self-designed, the quality of measurement needed particularly evaluation however, the indicators were drawn from a series of questionnaires verified by multiple studies in different regions, and also referred to the results of interviews and Delphi method, therefore, the questionnaire had good reliability and validity Fourthly, only the viewpoints of migrants were taken into account in this study; the perspectives of healthcare professionals were disregarded. Additionally, due to the point-in-time nature of the data, the study did not measure the impact of changes over time. Lastly, since all participants were sampled in Zhejiang Province, the results may not be representative in other regions. However, since Zhejiang Province is the pilot area of medical alliance, the form of medical alliance in Zhejiang Province is similar to other regions, so the results have certain reference value.

Above all, there is no doubt that establishing long-term relationships between migrants and healthcare professionals is key to further optimizing the coordination and continuity mechanisms. Consequently, it is worth further discussion that extensional research goals could focus on the targets and demands of healthcare services recipients rather than merely on those of the providers. Clearly, whether the main reason for poor continuity is the services provider or the patient and which is more dominant could both be probed in subsequent research. In addition, the influencing factor of the perceived continuity of migrants in our research such as the external environment, features of the population, health behavior, and health outcome, can be associated with the framework of the Andersen behavioral model. This approach will further ensure that the impact of comprehensive factors related to migrants (such as social integration, technical factors, environmental, and cultural factors)on perceived continuity can be validated. Ultimately, this study can expand the sample size in domestic regions, then conduct a multi-group analysis and comparison to determine whether there is an inequality in this continuity of healthcare influence mechanism of migrants across regions. Hence, future studies should include an objective evaluation index, analyze migrants and providers to learn more about the factors that affect continuity of healthcare, and consider data collection from other districts.

## 6. Conclusion

The perception of migrant continuity was significantly improved by the PCIC practice in medical alliances. Accessibility and comprehensiveness mediated the positive correlation between coordination and continuity. In the developed region, we discovered that respect had a moderate mediating influence on the relationship between coordination and continuity through comprehensiveness. In addition, among migrants in the developing region, males and professionals experienced better service continuity. Therefore, measures to promote the continuity of healthcare through PICI in medical alliances should be paid attention to, including the coordination, accessibility, comprehensiveness, and respect.

## Data availability statement

The original contributions presented in the study are included in the article/Supplementary material, further inquiries can be directed to the corresponding authors.

## Ethics statement

The studies involving human participants were reviewed and approved by the Ethics Committee of Hangzhou Normal University. Written informed consent to participate in this study was provided by the participants' legal guardian/next of kin.

## Author contributions

HZ: conceptualization, methodology, and software. YW and WS: formal analysis, writing—original draft preparation, and validation. WL and ZH: investigation. XH and TS: review. MW: data collection. ZH: writing. ZH, XH, and TS: supervision. SC: writing—review and editing. All authors approved the final manuscript.
